# Beyond the “string of beads”: case-based exploration of diagnostic pitfalls and solutions in reversible cerebral vasoconstriction syndrome

**DOI:** 10.1186/s10194-025-01978-5

**Published:** 2025-04-28

**Authors:** Seung Ae Kim, Eung Yeop Kim, Shuu-Jiun Wang, Mi Ji Lee

**Affiliations:** 1 Department of Neurology, Seoul National University Hospital, Seoul National University College of Medicine, 101 Daehak-ro, Jongno-gu, Seoul, 03080 Republic of Korea; 2 Graduate School of Translational Medicine, Seoul National University College of Medicine, Seoul, Republic of Korea; 3 Department of Radiology, Samsung Medical Center, Sungkyunkwan University School of Medicine, Seoul, Republic of Korea; 4 Department of Neurology, Neurological Institute, Taipei Veterans General Hospital, Taipei, Taiwan; 5 College of Medicine, National Yang Ming Chiao Tung University, Taipei, Taiwan; 6 Brain Research Center, National Yang Ming Chiao Tung University, Taipei, Taiwan

**Keywords:** Reversible cerebral vasoconstriction syndrome, Thunderclap headache, Angiography, Imaging artifacts, Cerebral vasoconstriction, Differential diagnosis, High-resolution vessel wall MRI, Blood-brain barrier imaging

## Abstract

**Background:**

The diagnosis of reversible cerebral vasoconstriction syndrome (RCVS) is challenging due to its varied clinical manifestations and imaging findings. While it typically presents with a sudden, severe thunderclap headache and multifocal constriction of the cerebral arteries, the wide spectrum of radiological presentations may complicate the diagnosis.

**Main Body:**

This review presents a series of cases that show both typical and atypical presentations of RCVS. Typical cases show the characteristic “string of beads” pattern on angiography, which usually resolves within 3–6 months. However, diagnostic challenges arise when angiography appears normal in the early stages or when imaging artifacts obscure the findings. In addition, the variability in vasoconstriction patterns and the need for a differential diagnosis further complicate the accurate identification. These cases highlight the importance of considering RCVS in patients with recurrent thunderclap headaches, even when the initial imaging is inconclusive. Recognizing these challenges and the variability in presentation, along with the use of high-resolution vessel wall MRI and blood-brain barrier imaging, can improve diagnostic accuracy and improve patient outcomes.

**Conclusion:**

The diagnosis of RCVS requires careful integration of clinical evaluation and advanced imaging techniques, with particular attention to radiological findings that can guide accurate diagnosis and management. Despite challenges, such as normal early stage angiography and imaging variability, maintaining a high suspicion of RCVS is essential, especially in patients with recurrent thunderclap headaches.

## Background

Reversible cerebral vasoconstriction syndrome (RCVS) has been introduced as a clinical entity in 2007 [1]. The initial diagnostic criteria included recurrent thunderclap headaches, multifocal cerebral artery vasoconstriction with reversibility within 12 weeks on angiography, normal cerebrospinal fluid findings, and the exclusion of other causes [1]. Multifocal segmental vasoconstriction of RCVS often accompanies with vasodilation and can be seen with a “string of beads” appearance on angiography, which has traditionally been considered as characteristic for the primary angiitis of the central nervous system (PACNS) [1–3]. Thus, the initial diagnostic criteria seem to emphasize the differentiation from PACNS relying on cerebrospinal fluid analysis and the reversibility of angiographic abnormalities.

However, growing clinical experience has revealed that RCVS may encompass a broader spectrum of clinical and radiological presentations than initially recognized. While the International Classification of Headache Disorders, 3rd edition provides diagnostic criteria specifically for the headaches attributed to RCVS, it does not offer a diagnostic framework for RCVS itself [4]. Given the dynamic nature of vascular changes in RCVS, understanding the full spectrum of its radiological manifestations is essential for accurate diagnosis and differentiation from other conditions.

This paper focuses on the radiological aspects of RCVS, aiming to comprehensively review the diagnostic pitfalls, provide a full spectrum of angiographic findings observed in real-world cases, and make suggestions for the differential diagnosis.

## Typical reversible cerebral vasoconstriction syndrome

RCVS typically presents as repetitive sudden and severe thunderclap headaches and reversible multifocal constriction of the cerebral arteries [1, 2, 4, 5]. Angiographic imaging findings characteristic of RCVS is a “string of beads” or “sausage-on-a-string” pattern, which resolved within 3–6 months [4]. These initial cases illustrate the classic presentation of RCVS, emphasizing the critical role of identifying this imaging signature alongside clinical symptoms for an accurate diagnosis.

### Case 1

A 37-year-old female at 38 weeks of gestation experienced labor pain and took a shower before heading to the hospital. As soon as the water hit her body, she was struck by a sudden onset, excruciating headache. After childbirth, the patient continued to experience recurrent thunderclap headaches. Her thunderclap headache was triggered by activities such as showering, bending, and urination.

**Fig. 1:**
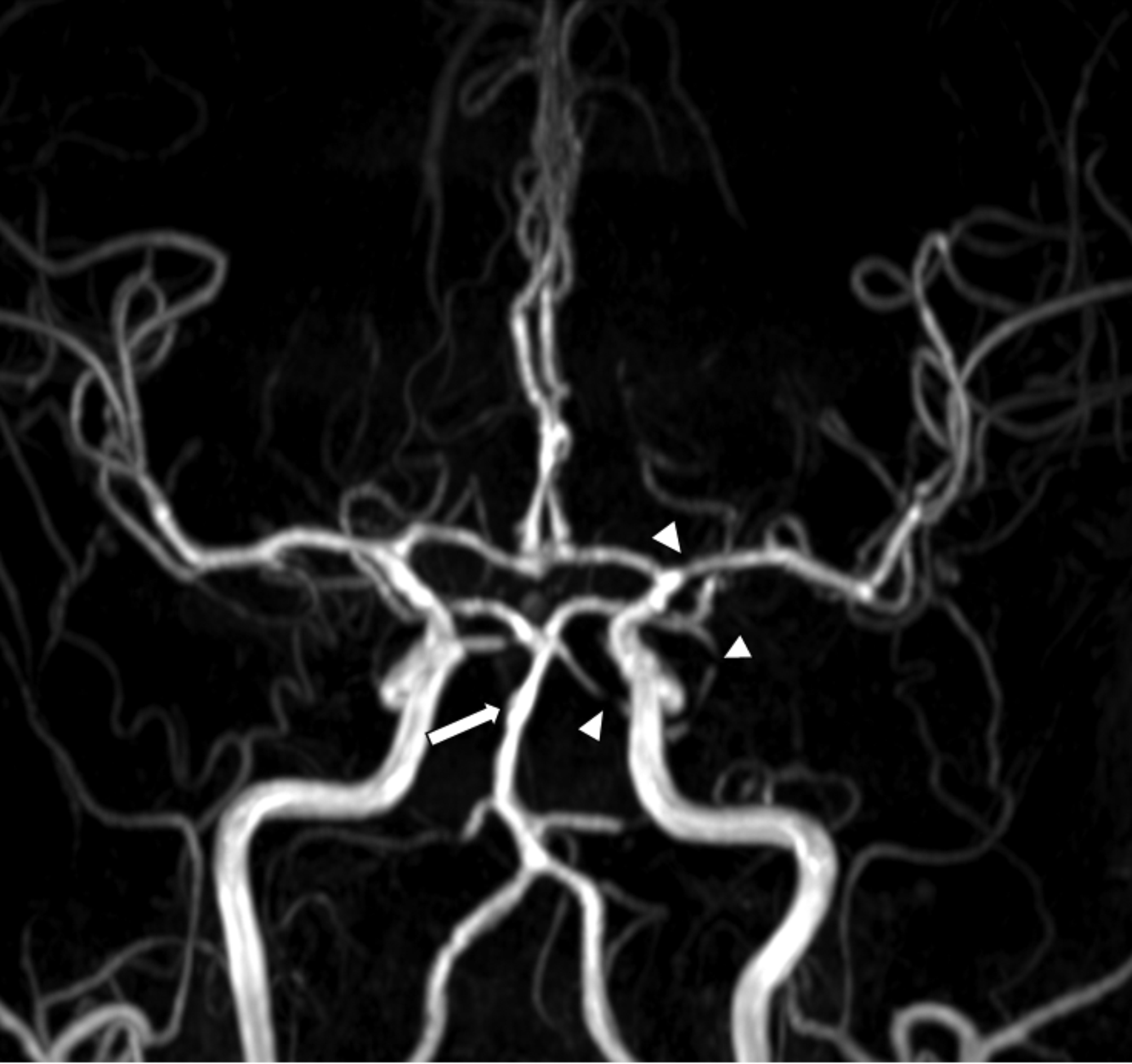
Typical steno-dilatation in reversible cerebral vasoconstriction syndrome: case 1. Time-of-flight magnetic resonance angiography (TOF MRA) image taken 17 days after onset in a pregnant woman with RCVS showed segmental stenosis (arrowheads: highlighting only selected regions for clarity) in multiple cerebral arteries and post-stenotic dilatation in the basilar artery (arrow) and other cerebral arteries, presenting the characteristic “string of beads” or “sausage-on-a-string” pattern of RCVS

Angiographic imaging revealed segmental stenosis and post-stenotic dilatation in multiple cerebral arteries, presenting a characteristic pattern of alternating constriction and dilation, similar to a “string of beads” or “sausage-on-a-string” pattern (Fig. 1). Based on the clinical presentation and imaging findings, the diagnosis of RCVS was made.

### Case 2

A 56-year-old female came to the clinic after being startled by a fire alarm in her apartment 8 days prior. The following morning, the patient experienced a sudden and severe thunderclap headache while brushing her teeth. The headache recurred multiple times thereafter and was notably triggered by actions such as coughing, bending over, tying her hair, and speaking in tense situations.

**Fig. 2:**
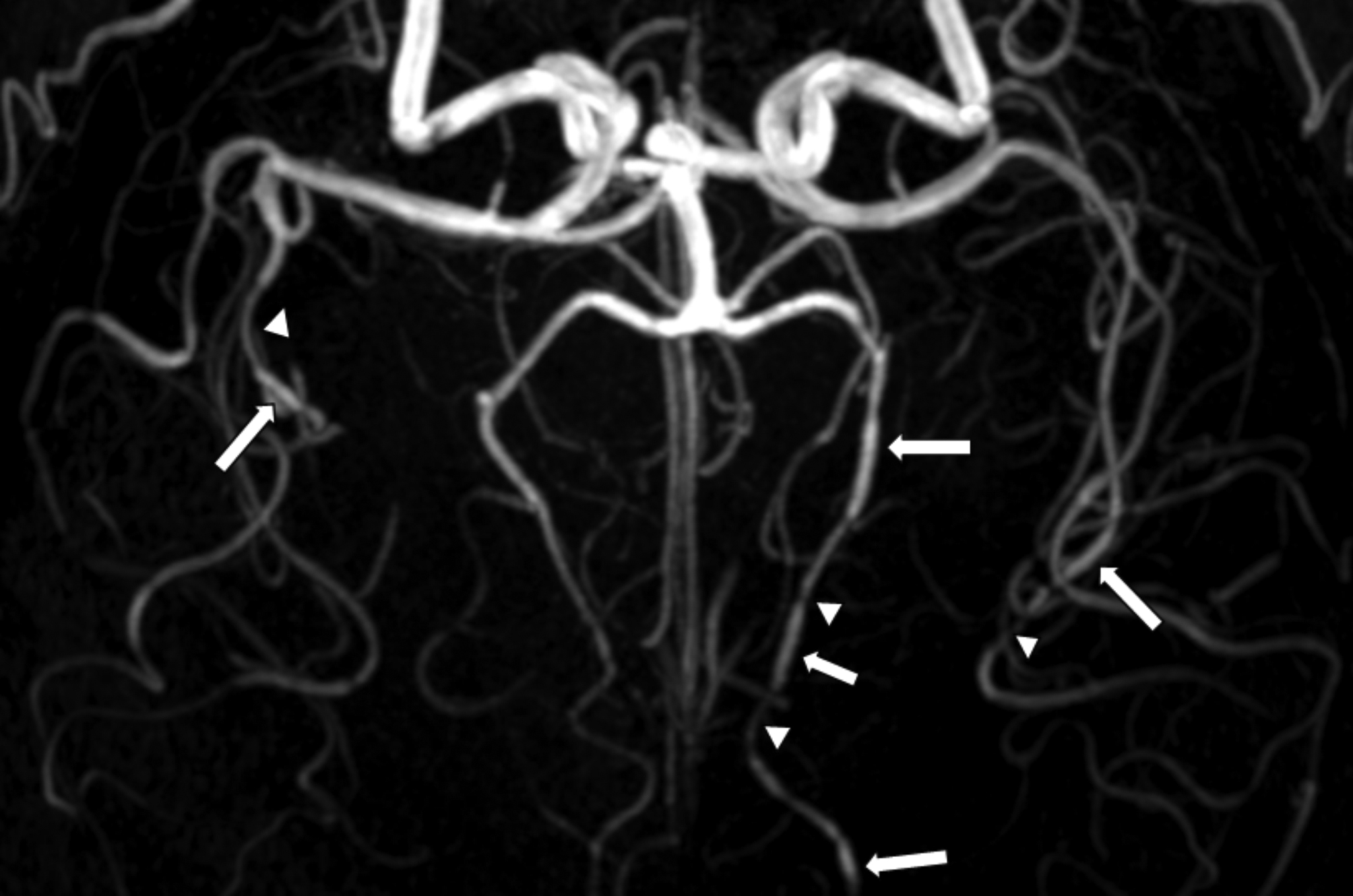
Typical steno-dilations in reversible cerebral vasoconstriction syndrome: case 2. Multiple segmental stenoses (arrow heads) and dilations (arrows) in the distal middle cerebral arteries (MCAs) and posterior cerebral arteries (PCAs) in a 56-year-old patient with RCVS experienced a thunderclap headache triggered by brushing teeth, coughing, bending, tying hair, and speaking in tense situations. Only representative regions are marked for clarity

The imaging revealed multiple segmental stenoses and dilatations, predominantly in the distal middle and posterior cerebral arteries (Fig. 2). Therefore, the patient was diagnosed with RCVS.

## Diagnostic challenges and variability of magnetic resonance angiography

Although the typical cases presented above are straightforward, several scenarios complicate the diagnosis of RCVS. Challenges include normal angiography in the early stages, artifacts and sensitivity issues of angiographic imaging modalities, and variability in the distribution and degree of vasoconstriction. These examples underscore the importance of comprehensive clinical evaluation. Even when imaging is normal or atypical, RCVS should remain a differential consideration, especially when patients present with recurrent thunderclap headaches. In this section, we will further elucidate these diagnostic nuances with case studies.

### Normal angiography

Angiographic imaging, including MR, CT, and catheter angiography, can reveal normal findings within the first week of clinical onset [6, 7]. In such cases, patients with recurring thunderclap headaches but with normal angiograms should be considered for RCVS. The ICHD-3 called this condition “probable RCVS,” where a patient shows a typical clinical manifestation of RCVS but normal angiographic findings [4]. This challenge underscores the need for clinical vigilance and a comprehensive evaluation beyond imaging alone, particularly in the early stages of RCVS, when angiography may not reveal characteristic findings.

#### Case 3

**Fig. 3:**
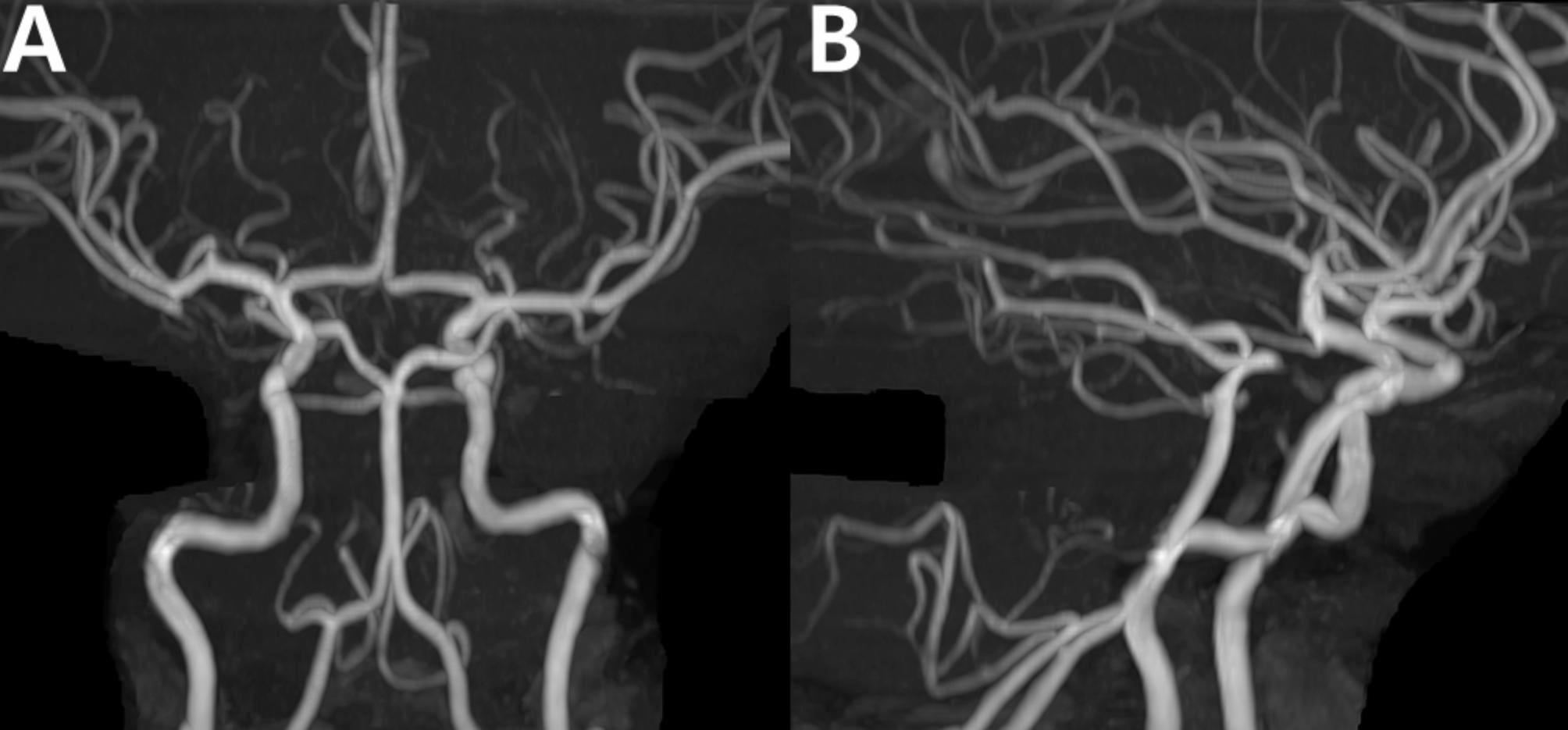
Reversible cerebral vasoconstriction syndrome with normal brain magnetic resonance angiography findings. Brain MRA of a 31-year-old patient with recurrent thunderclap headaches triggered by typical precipitants, which did not show significant abnormalities

A 31-year-old female experienced a thunderclap headache 1 month before her visit to the clinic. Two weeks before admission, after returning from vigorous exercise, the patient awoke the next morning with another thunderclap headache. The headache was exacerbated by actions such as standing up from a seated position, getting up from lying down, sneezing, and blowing one’s nose. She reported feeling a pulsating sensation in her head that coincided with her heartbeat, accompanied by pain of 8 out of 10 on the numeric rating scale, lasting for approximately 5 min. With the impression of RCVS, brain magnetic resonance imaging (MRI) and MRA were performed, but did not reveal significant findings (Fig. 3). A tentative diagnosis of probable RCVS was made. The patient was treated with nimodipine and did not experience significant headaches thereafter. The diagnosis of probable RCVS was finally made after 3 months of observation.

### Magnetic resonance angiography artifacts and sensitivity issues

MRA and CTA are both effective for evaluating large vessels in RCVS [8]. CTA uses iodinated contrast media to directly visualize vascular structures, providing accurate anatomical representation of vessel morphology [9–11]. However, optimal visualization of distal vessels on CTA depends on precise contrast bolus timing, which can be inconsistent in clinical settings. These factors may lead to variability in the quality of imaging, particularly in the visualization of distal vasculature. Additionally, CTA is often limited by its inability to comprehensively evaluate the vessel wall or brain parenchyma, as well as the risk of nephrotoxicity and radiation exposure associated with the use of iodinated contrast agents.

In contrast, time-of-flight (TOF)-MRA visualizes intracranial arteries through flow-related enhancement rather than contrast media, eliminating the risks associated with nephrotoxicity and radiation exposure [12]. Although it is susceptible to flow-related artifacts and potential signal loss in regions with complex or slow flow patterns, TOF-MRA offers standardized imaging parameters that enhance its reproducibility [8, 13, 14]. TOF-MRA also has the advantage of detecting hyperintensity in the vessel wall, aiding in the diagnosis of conditions such as arterial dissection, and can be further enhanced by combining it with complementary sequences such as Susceptibility-Weighted Imaging, Vessel Wall Imaging or Contrast-Enhanced Fluid-Attenuated Inversion Recovery. These attributes make MRA widely used as a first-line diagnostic modality in clinical practice.

While both modalities are influenced by technical factors during image acquisition, the comprehensive evaluation capabilities and safety profile of TOF-MRA make it the preferred first-line diagnostic modality for RCVS. As noted, there are several factors influencing TOF-MRA acquisition. In this section, we will review potential issues in interpreting TOF-MRA that can lead to misdiagnosis of RCVS.

#### Stair-step artifact

**Fig. 4:**
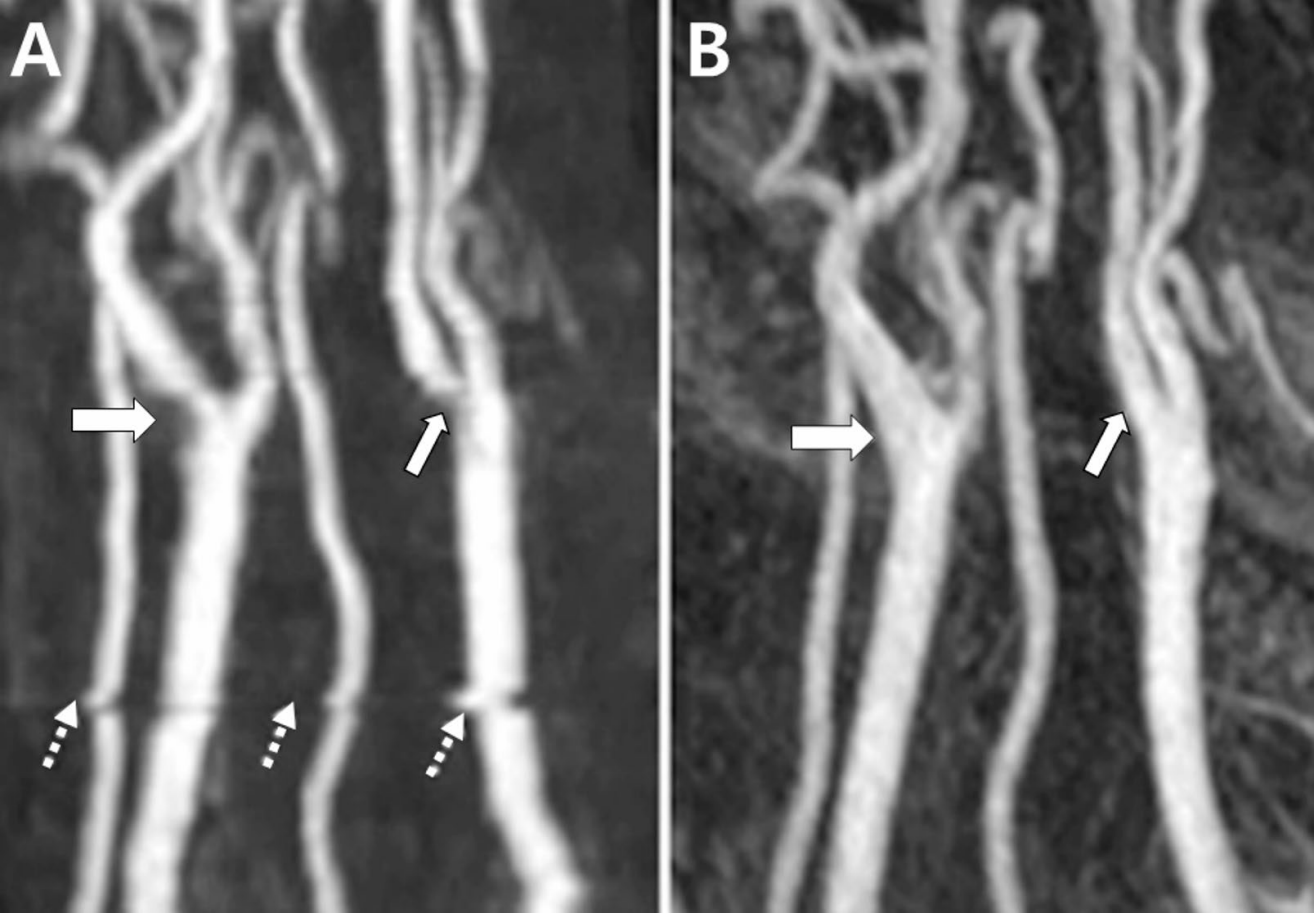
Stair-step artifacts. (**A**) Pseudo-stenosis caused by stair-step artifacts in 2D TOF MRA is observed in the right internal carotid artery (ICA) (thick arrow), left ICA (thin arrow), bilateral vertebral arteries, and left common carotid artery (dotted arrows). (**B**) In the contrast-enhanced MRA of the same patient, no artifacts are seen in the vessels indicated in (**A**). Figure adapted from McKinney AM: Artifacts of the Craniocervical Arterial System on MRI. In: *Atlas of Normal Imaging Variations of the Brain*,* Skull*,* and Craniocervical Vasculature*. Cham: Springer International Publishing; 2017: 1261–1291, under the terms of the Creative Commons Attribution License [16]

This artifact occurs on 2D TOF MRA where the slices are relatively thick, resulting in a pixelated appearance of obliquely oriented vessels. This can mislead the interpretation of the vessel structure, making it appear as steplike patterns in the image [9, 15]. The vessels appear segmented and pixelated, which can interfere with the accurate diagnosis (Fig. 4).

#### In-plane saturation artifact

**Fig. 5:**
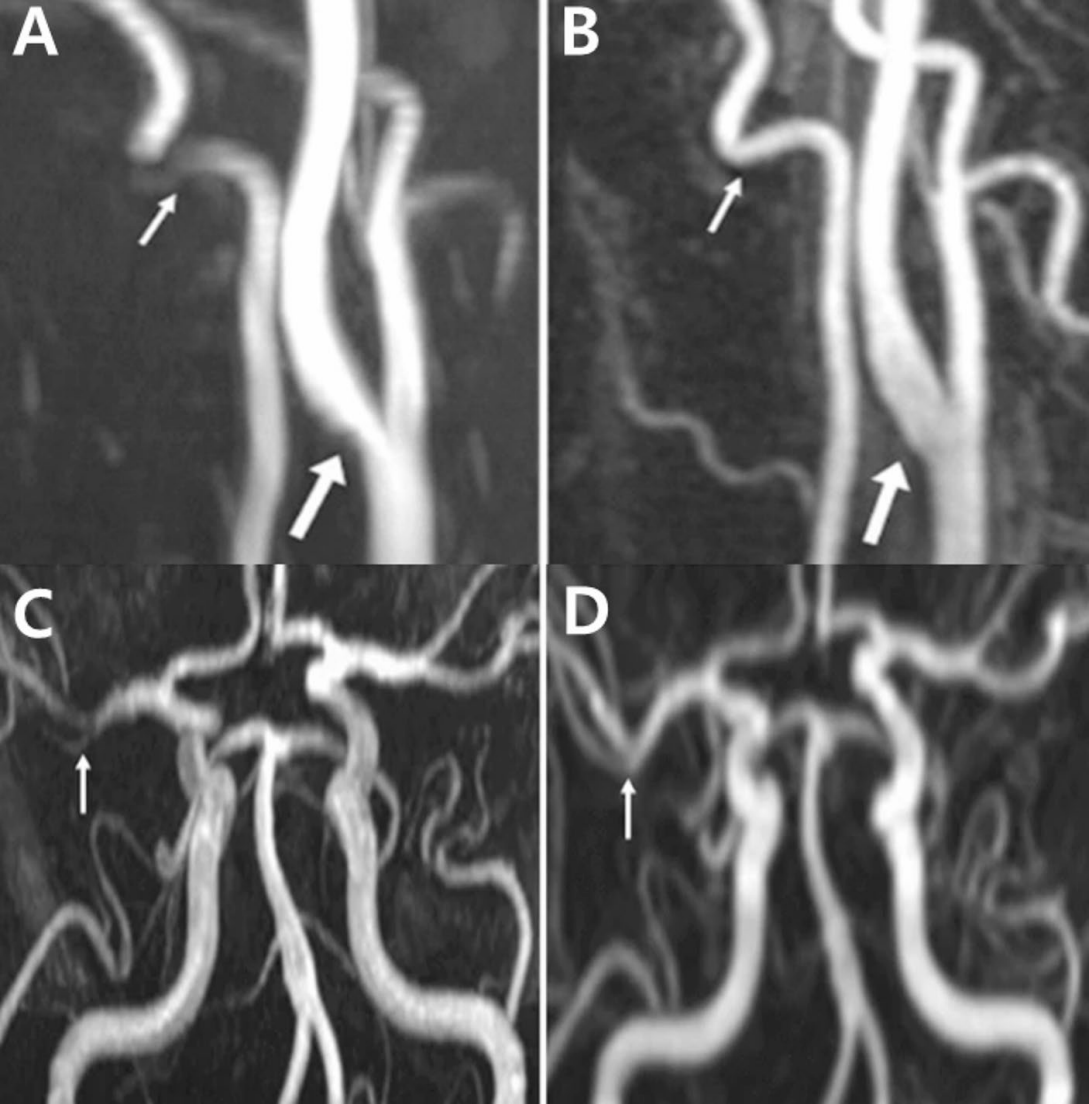
In-plane saturation artifact. (**A**) Pseudo-stenosis in the ICA (thick arrow) and in-plane saturation artifacts in the VA (thin arrow) are observed in a 2D TOF MRA. (**B**) The contrast-enhanced MRA shows normal findings in the previously observed areas. (**C**) A pseudo-stenosis that appears as an artificial defect in the right MCA (arrow) is evident on a 3D TOF MRA. (**D**) However, this defect is not seen in the contrast-enhanced MRA image. Figure adapted from McKinney AM: Artifacts of the Craniocervical Arterial System on MRI. In: *Atlas of Normal Imaging Variations of the Brain*,* Skull*,* and Craniocervical Vasculature*. Cham: Springer International Publishing; 2017: 1261–1291, under the terms of the Creative Commons Attribution License [16]

TOF MRA achieves a high intravascular signal using the inflow of fresh unsaturated blood, with the signal being most intense when the direction of blood flow is perpendicular to the imaging plane​ [17]. However, when vessels run within the plane, blood can become saturated, similar to stationary tissues, leading to reduced signals [18] (Fig. 5).

#### Venetian blind artifact

**Fig. 6:**
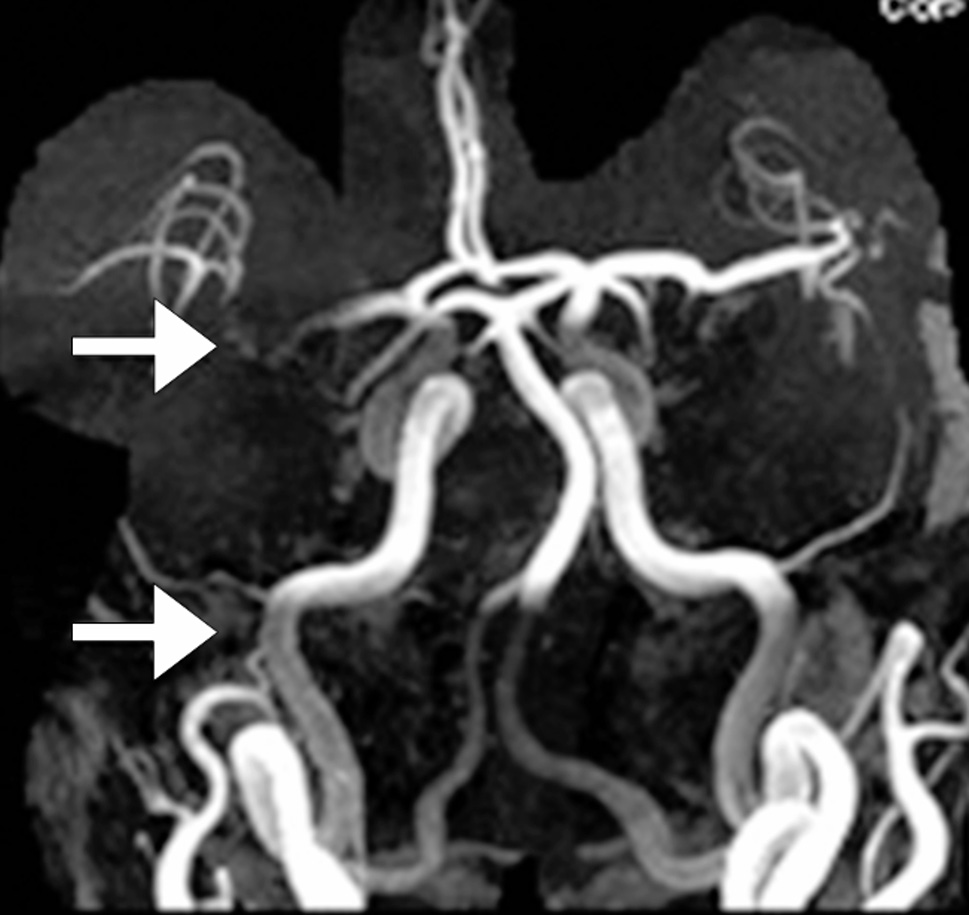
Venetian blind artifacts in 3D TOF imaging resulting from signal loss due to repeated radiofrequency pulses, particularly at slab edges (arrows). Figure adapted from Sayah A, Mamourian AC: Flow-Related Artifacts in MR Imaging and MR Angiography of the Central Nervous System. *Neurographics* 2012, 2(4):154–162, under the terms of the Creative Commons Attribution License [19]

In 3D TOF imaging, the signal intensity decreases as a result of repeated radiofrequency pulses as the protons move through the imaged volume. This loss is most noticeable at the slab edges. When the slabs are combined, it creates a “Venetian blind” effect, with low signal intensity at section boundaries [19] (Fig. 6).

#### Visualization limitations in distal vessels

TOF MRA is effective for imaging large- and medium-sized arteries, but faces challenges with small distal vessels [20]. Loss of signal from repeated radiofrequency pulses reduces the visibility and structural clarity of these vessels [20, 21]. The real-world challenges are exemplified in Case 4.

##### Case 4

A 58-year-old female stood outside for an extended period on a cold day, 6 days before experiencing a sudden thunderclap headache. Subsequently, the headache recurred several times and was notably triggered by urination and defecation.

**Fig. 7:**
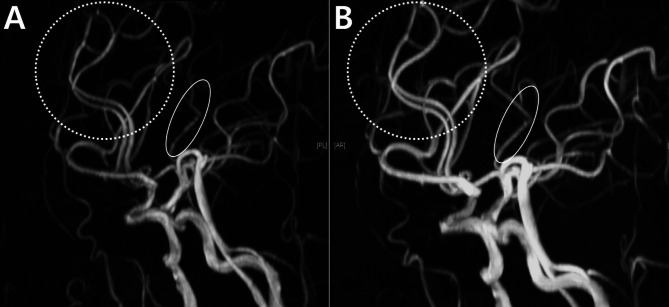
Reversible cerebral vasoconstriction syndrome with ambiguous findings: case 4. (**A**) An initial MRA performed 6 days after onset shows uncertainty between distal artery visualization issues and possible stenosis, particularly in the anterior cerebral arteries (ACAs) (dotted circles) and the MCA (oval). (**B**) Follow-up MRA 3 months later reveals distal vessel dilation in the same ACAs (dotted circles) and MCA (oval), suggesting RCVS

During the MRA examination, 6 days after the onset of the symptoms, it was challenging to distinguish between normal findings and artifacts (Fig. 7A). However, 3 months later, distal vessel dilation became evident, leading to the diagnosis of RCVS (Fig. 7B). This case highlighted the diagnostic limitations of MRA, particularly its reduced sensitivity and specificity for distal vessels, which can obscure early signs of RCVS. This case provides insight into the challenges in diagnosing RCVS, particularly in its early stages, and highlights the importance of follow-up imaging to detect evolving vascular changes indicative of the condition.

## Diverse spectrum of degree and distribution of Vasoconstriction in Reversible Cerebral Vasoconstriction Syndrome

As discussed above, the typical radiological manifestation of RCVS is the presence of multiple segmental stenoses and dilatations in the cerebral arteries, often described as a “string of beads” or “sausage-on-a-string” appearance and is observed most prominently between 1 and 3 weeks accompanied by recurrent thunderclap headaches [1, 2, 4, 22]. However, RCVS can be captured with different degrees and distributions of vasoconstriction, due to different severities of disease activity or the timing of the imaging.

### Case 5

**Fig. 8:**
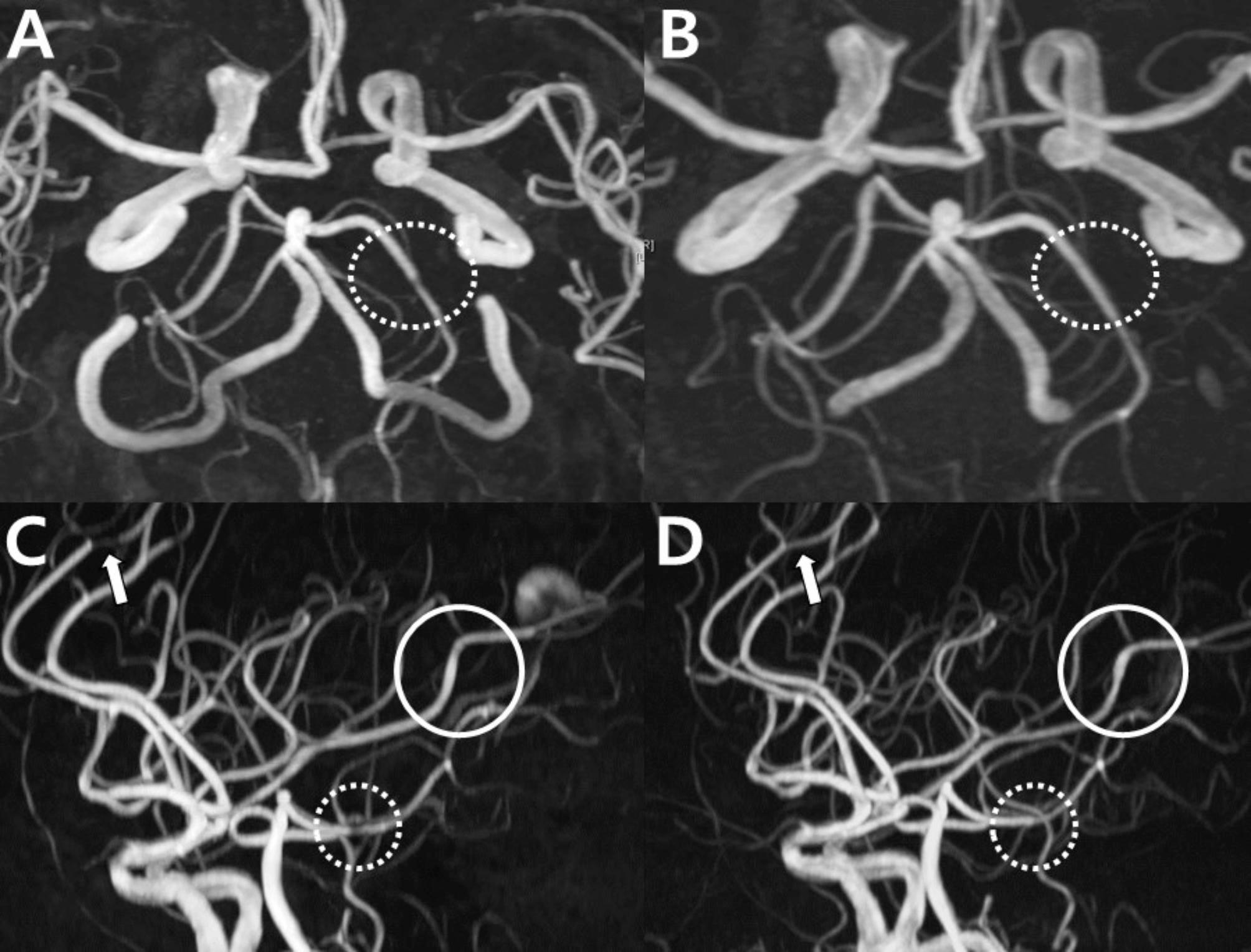
Rapid reversibility of stenosis and new focal dilation in reversible cerebral vasoconstriction syndrome: Case 5. (**A**) Initial MRA with focal stenosis in the left PCA (dotted circle). (**B**) Follow-up MRA in 2 days showing normalized stenosis (dotted circle). (**C**) Initial MRA suspicious for stenosis vs. artifact in the right anterior (arrow) and left PCAs (dotted circle). (**D**) The follow-up MRA shows normalization of the right anterior (arrow) and left PCAs (dotted circle) and a focal dilation in the left MCA (circle) newly developed

A 67-year-old female experienced a sudden thunderclap headache while bending over to pick up a fallen object 10 days prior, which resolved spontaneously. MRA revealed a single focal stenosis of the left PCA (Fig. 8A). Since the vasoconstriction involved only a single segment, the diagnosis of RCVS could not be confirmed, and the differential diagnoses included atherosclerosis and dissection. Two days later, the patient returned to the hospital with sudden diplopia, right-hand weakness and transient aphasia, each lasting about 10 min. These neurological symptoms occurred alongside recurrent thunderclap headaches. Follow-up imaging revealed normalization of focal segmental stenosis in the left PCA (Fig. 8B). The diagnosis of RCVS was supported by the rapid reversibility of stenosis, which helped exclude other diagnoses. In this case, a new focal dilation was observed in the right ACA and the left MCA (Fig. 8D), which was not apparent in the initial MRA (Fig. 8C). This case illustrates that RCVS can manifest itself as a single-segment vasoconstriction or dilatation.

### Case 6

**Fig. 9:**
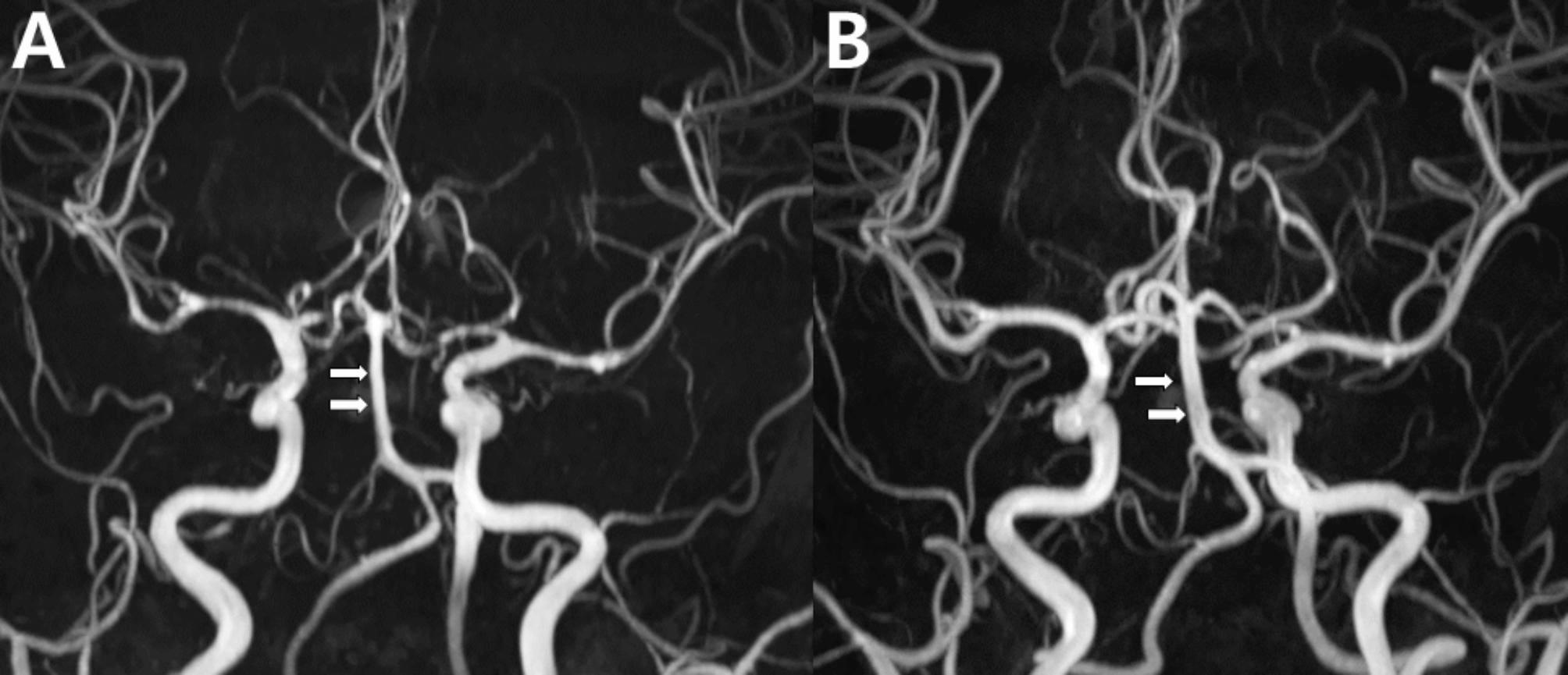
Diffuse narrowing of the basilar artery as a reversible cerebral vasoconstriction syndrome phenomenon: case 6. (**A**) Multifocal narrowing of the bilateral M1, A1, and A2 segments is observed in the initial time-of-flight MR angiography of a 30-year-old patient with postpartum RCVS. In this imaging, the basilar artery (arrows) appeared normal. (**B**) The follow-up imaging obtained 3 months later showed normalization of the overall vessels. The diameter of the basilar artery increased compared to the initial imaging (arrows), indicating that there is a diffuse narrowing in the basilar artery, which can be missed if assessed cross-sectionally

A 30-year-old female, 7 days postpartum, experienced her first headache, which gradually began with only moderate intensity. Her MRA revealed multifocal narrowing of the bilateral M1, A1, and A2 segments (Fig. 9A). Although she did not have a typical thunderclap headache, the diagnosis of RCVS was made once her follow-up imaging showed normalization of the vasoconstrictions. Interestingly, the basilar artery, which initially appeared normal, showed an increase in diameter in the follow-up imaging (Fig. 9B), suggesting the possibility that she initially had a “diffuse narrowing” in the basilar artery (Fig. 9A). This observation raises the possibility that cases deemed “angiographically normal” could harbor such a diffuse narrowing, which warrants a more cautious evaluation. The diffuse narrowing of the vessel can initially be overlooked. Follow-up imaging can help, even in patients with normal-looking MRA.

## Reversible cerebral vasoconstriction syndrome-mimicking clinical conditions

### Intracranial arterial atherosclerosis

#### Case 7

**Fig. 10:**
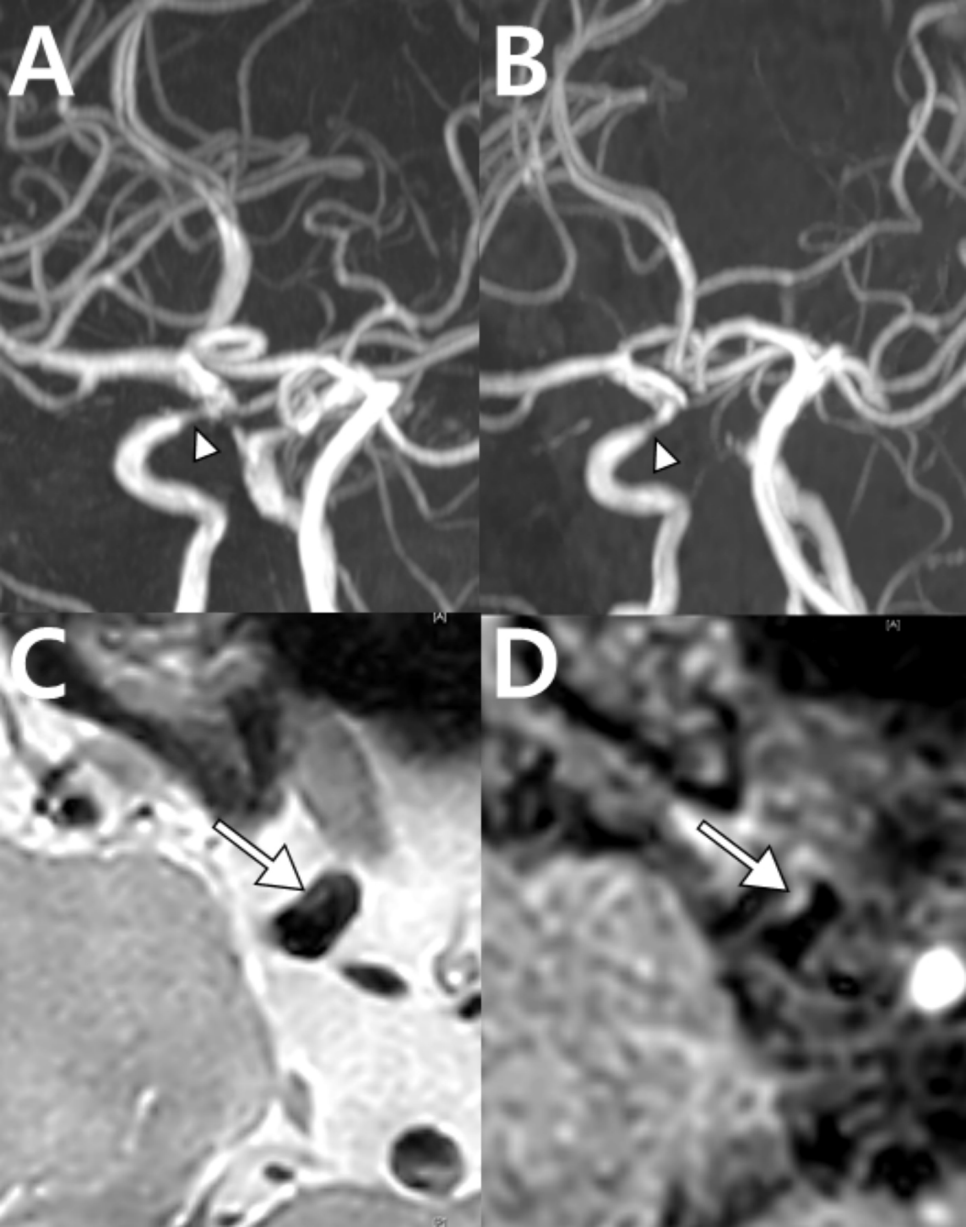
Mimickers of reversible cerebral vasoconstriction syndrome: focal intracranial atherosclerosis in a patient with reversible cerebral vasoconstriction syndrome (case 7). (**A**) Multifocal stenosis is observed in the ACA, MCA, and most prominently in the right distal ICA (arrowhead) in the initial time-of-flight MR angiography. (**B**) Six months later, overall vasospasm improved, except for the right distal ICA (arrowhead). (**C, D**) High-resolution vessel wall MRI (C, T2 weighted image; D, T1 post-contrast image) show an atherosclerotic plaque in the right distal ICA (arrow)

A 51-year-old female visited our headache clinic 7 days after experiencing a thunderclap headache while swimming. The headache resolved and did not recur after stopping swimming. MRA revealed multifocal stenosis and dilatation of the ACA [23], MCA, and distal ICA (Fig. 10A). Vasospasm improved after 6 months, except for a lesion in the right distal ICA (Fig. 10B). High-resolution vessel wall MRI revealed an atherosclerotic plaque in the right distal ICA (Fig. 10C and D). Therefore, the right distal ICA was determined to have atherosclerosis probably prior to the onset of RCVS, while the vasoconstrictions in the other vessels were confirmed as RCVS.

#### Case 8

**Fig. 11:**
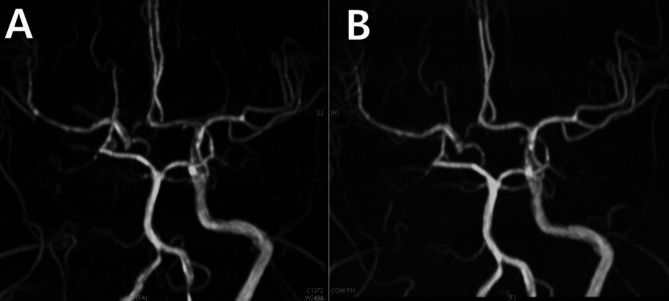
Mimickers of reversible cerebral vasoconstriction syndrome: asymptomatic atherosclerosis (case 8). (**A**) MR angiography shows right ICA occlusion with multifocal stenosis in the distal M1, M2, M3, A3, and V4 segments in a 74-year-old patient with clinically suspected RCVS. (**B**) There is no significant change 1 year after onset. The diagnosis of asymptomatic intracranial atherosclerosis and probable RCVS (angiogram-negative) is made

A 74-year-old female came to the emergency room with a thunderclap headache while defecating. Her headache recurred three times, when bending or defecating. Right ICA occlusion with multifocal stenosis was observed in the distal M1, M2, M3, A3, and V4 segments (Fig. 11A). Although clinical symptoms were consistent with RCVS and some minor narrowing appeared to have normalized, most of the arterial narrowing persisted for more than 1 year, indicating that these vasoconstrictions were of atherosclerotic origin (Fig. 11B). The patient was finally diagnosed with asymptomatic atherosclerosis and probable (angiogram-negative) RCVS (Fig. 11).

Intracranial atherosclerotic stenosis (ICAS) is an important differential diagnosis of RCVS due to its high prevalence. Asymptomatic ICAS is common in the general population (6–13%), with an even higher prevalence (9–65%) in hospital-based studies from Asian countries [24–26]. ICAS is one of the most common causes of stroke worldwide and is associated with substantial morbidity and mortality [25]. Differentiating between ICAS and RCVS is essential because they require different treatment strategies. Although the distinct feature of RCVS is its reversibility, serial imaging is required to document reversibility; consequently, a definitive diagnosis is delayed. High-resolution vessel wall MRI (HR-vwMRI) can prompt differential diagnosis between atherosclerosis and RCVS. The HR-vwMRI characteristics of ICAS include the presence of eccentric atheromas with various signal intensities, positive remodeling of the affected segment, and eccentric plaque enhancement [27]. Additionally, these plaques may show juxtaluminal T2 hyperintensity of the fibrous cap, a lipid-rich necrotic core, and thickening or remodeling of the vessel wall [27, 28]. In contrast, HR-vwMRI of RCVS can only show mild enhancement without positive remodeling or eccentric atheroma [28–31].

Another differential point is that RCVS rarely involves the ICA [32, 33], which is represented in the RCVS2 scoring system (see below for further discussion). Two scoring systems are used for the differential diagnosis of RCVS: RCVS2 and RCVS-TCH. The RCVS2 score is particularly helpful in distinguishing RCVS in patients with intracranial vasculopathies, while the RCVS-TCH score is designed to identify RCVS in patients with thunderclap headaches. The acronym RCVS2 denotes the variables: recurrent or single thunderclap headache (+ 5 points), carotid (intracranial) artery involvement (-2 points), vasoconstrictive trigger (+ 3 points), female sex (+ 1 point), and subarachnoid hemorrhage (+ 1 point) [32, 33]. An RCVS2 score, primarily based on CTA findings, of ≥ 5 is 90% sensitive and 99% specific to identify RCVS from other intracranial vasculopathies, while a score of ≤ 2 is 85% sensitive and 100% specific to rule out the condition [33, 34]. The RCVS-TCH score, which includes both patients evaluated with CTA and MRA, which includes recurrent thunderclap headache (+ 2 points), female sex (+ 3 points), blood pressure surge (+ 4 points), and triggering factors (multiple + 3 points, single + 2 points), suggests RCVS in patients with thunderclap headache at a cut-off point of ≥ 7, with a sensitivity of 80% and specificity of 97% [35]. The RCVS-TCH score has shown better performance compared to the RCVS2 score in identifying RCVS among patients with thunderclap headaches [35].

### Intracranial arterial dissection

#### Case 9

**Fig. 12:**
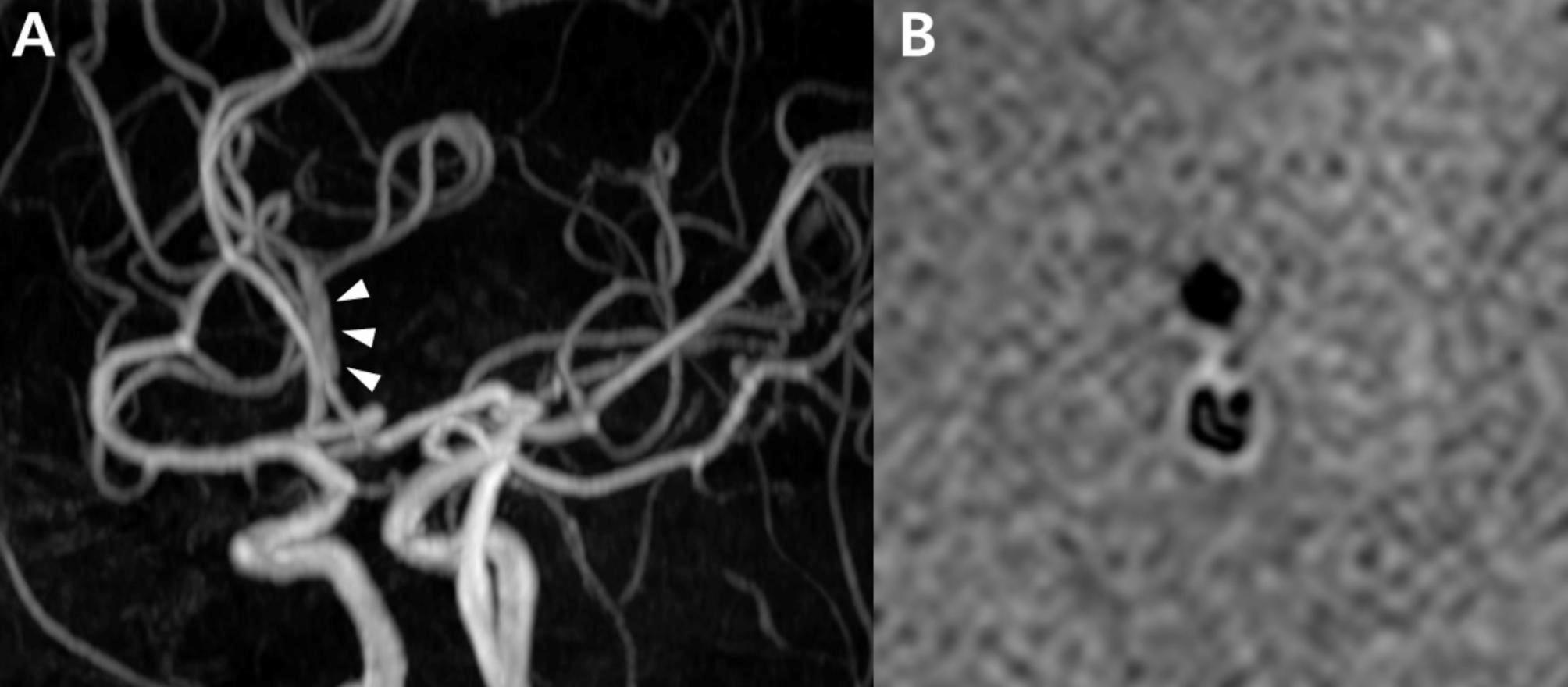
Mimickers of reversible cerebral vasoconstriction syndrome: Intracranial arterial dissection (case 9). The MRA shows a single dilation (arrowhead) in the left A2 segment in a patient with thunderclap headache (Case 9), who is ultimately diagnosed with dissection by (**B**) high-resolution vessel wall MRI revealing a dissecting flap

A 50-year-old female came to the emergency room after experiencing her first thunderclap headache while performing push-ups. MRArevealed a single dilation of the left A2 segment (Fig. 12A). Initially, RCVS was suspected; however, a high-resolution vessel wall MRIrevealed a dissecting flap, leading to a diagnosis of ACA dissection (Fig. 12). This case highlights the importance of consideringintracranial arterial dissection, especially in Asian populations, where it is relatively more common.

#### Case 10

**Fig. 13:**
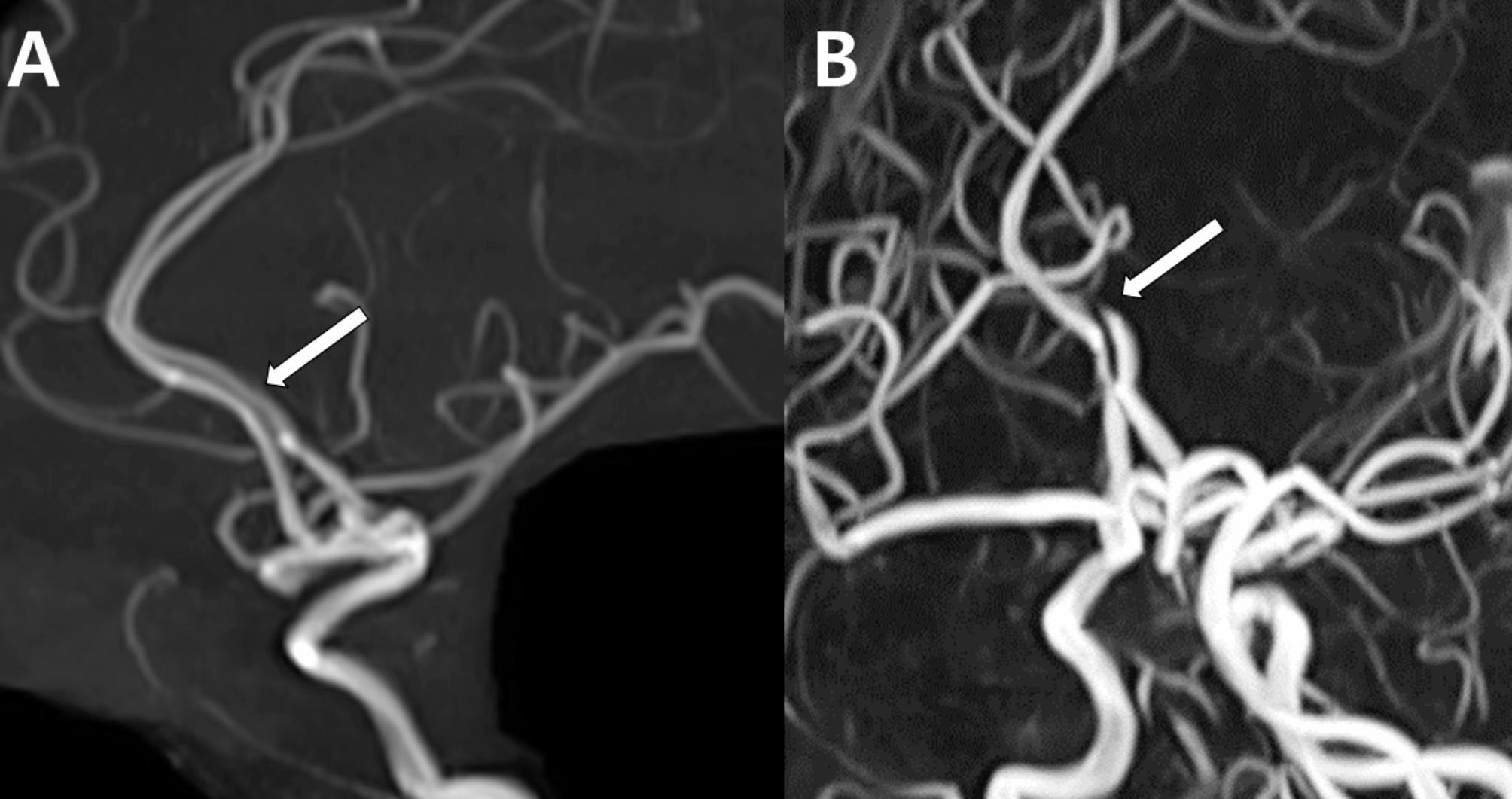
Reversible cerebral vasoconstriction syndrome resembling intracranial arterial dissection (case 10). (**A**) MRA taken 2 days after thunderclap headache showed normal findings in the left ACA (arrow). (**B**) However, a single flame-shaped occlusion in the left ACA (arrow) was observed 8 days after onset, suggesting RCVS rather than arterial dissection due to its dynamic imaging characteristics

A 47-year-old female experienced a thunderclap headache while performing push-ups 8 days before presentation. She initially visited another hospital 2 days after the onset, where cerebrospinal fluid (CSF) analysis was performed to rule out subarachnoid hemorrhage (SAH) but showed normal findings. MRA revealed no significant abnormalities (Fig. 13A), and she was diagnosed with stress-induced headache and discharged without further intervention. However, she experienced another thunderclap headache on day 8 after onset and visited our headache clinic. Imaging on day 8 revealed a single flame-shaped occlusion in the left ACA, leading to considerations of an arterial dissection (Fig. 13B). However, when comparing the MRI/MRA results of the initial hospital visit, normal findings were observed on day 2 after the onset (Fig. 13A). This dynamic change suggested RCVS rather than dissection.

Intracranial dissection and RCVS are important considerations in the differential diagnosis of patients with thunderclap headache. Intracranial dissection typically involves a single artery in approximately 70−80% of cases, while RCVS usually affects large-to medium-sized cerebral arteries in various vascular regions, leading to multisegmental constriction [1, 33, 36]. Intracranial dissection should be considered when there is arterial occlusion and recanalization with aneurysmal dilation, fusiform or irregular aneurysmal dilation at a non-branching site, or long irregular stenosis with double lumen, intramural hematoma, intimal flap, rapid changes, or focal stenosis [23, 37]. In East Asian studies, where most cases were reported due to recruitment from neurosurgery or neurointervention departments, intracranial artery dissection comprised 67–78% of cervicocephalic artery dissections [38, 39]. In South Korea, intracranial dissection accounts for approximately 1 in 4 cases of idiopathic MCA stenosis in young adults [40]. Furthermore, intracranial dissection was the most common cause of isolated ACA infarction in a study conducted in Japan [41]. Therefore, HR-vwMRI can aid in the differential diagnosis of intracranial arterial dissection and RCVS. Intracranial arterial dissection typically presents with eccentric luminal narrowing, intimal flaps, and intramural hematomas [42]. Among these findings [43, 44], intimal flaps (42−91.4% of cases) and intramural hematomas (23.1−61% of cases) are pathognomic for arterial dissection [44–46]. It should be noted that arterial dissection can trigger RCVS, and several reports document the coexistence of RCVS and dissection, with one study finding that 12% of RCVS cases and 7% of cervical artery dissection cases had both conditions​ [47–50].

### Moyamoya disease

#### Case 11

A 30-year-old female experienced a recurrent transient ischemic attack on the second postpartum day. These episodes involved temporary right arm paralysis lasting 10–30 min. In particular, she did not experience any thunderclap headache, but a mild headache (NRS, 3−4) following the ischemic attacks.

**Fig. 14:**
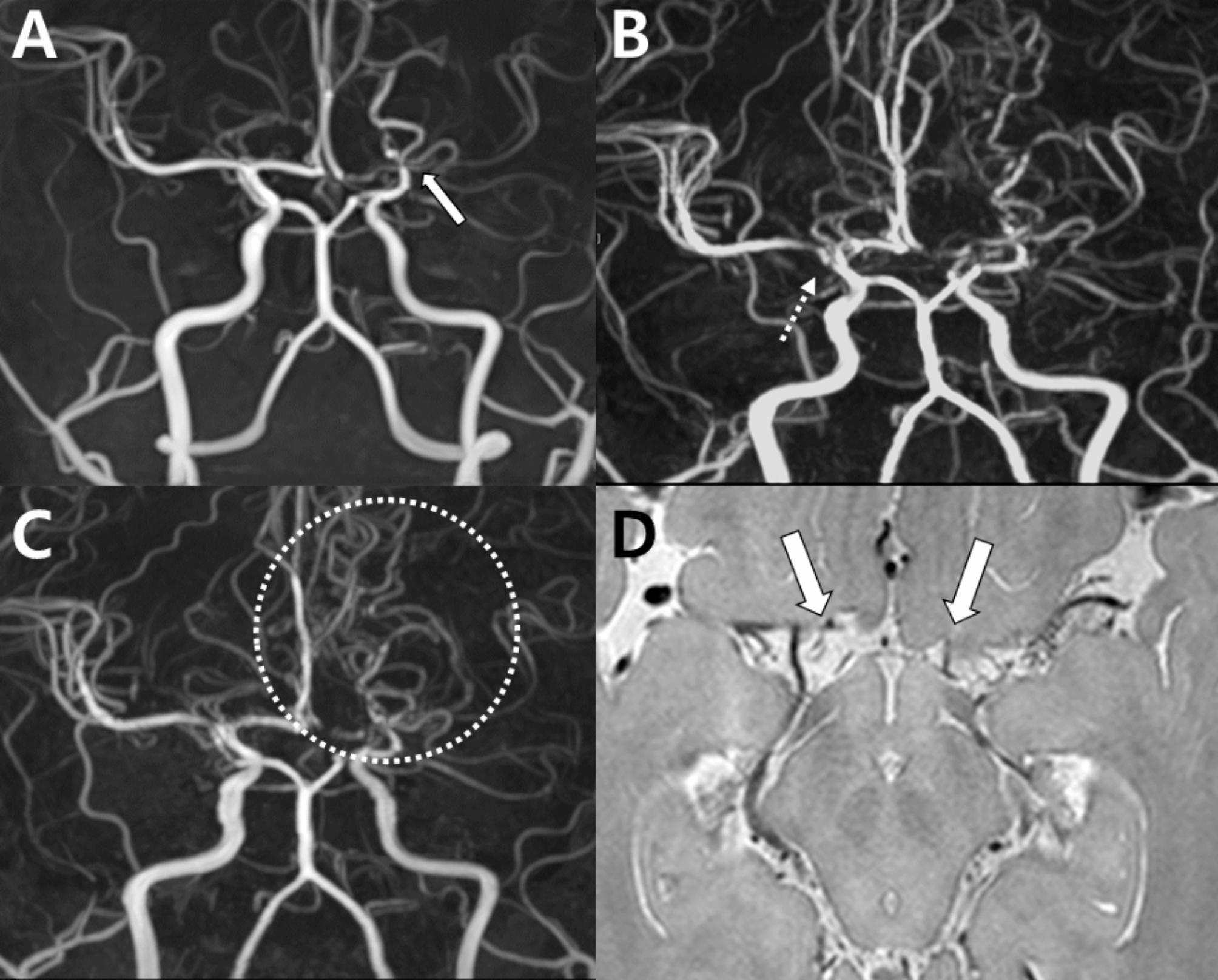
Mimickers of reversible cerebral vasoconstriction syndrome: unilateral moyamoya disease with rapid progression to bilateral disease (case 11). (**A-C**) Serial MRA angiographic imaging in a 30-year-old postpartum patient with recurrent transient ischemic attacks, right arm paralysis, and mild headache. (**A**) Initial imaging on the first day of onset shows isolated left distal ICA occlusion (arrow). (**B**) Subsequent imaging at 8 days after onset revealed rapid progression to right distal ICA stenosis (dotted arrow) along with left distal multifocal stenosis and a string-of-beads pattern. (**C**) At 1 year, no reversal of the bilateral distal ICA steno-occlusion or the vasoconstriction in the left distal arteries with formation of basal collateral vessels (dotted circle) were observed. (**D**) High-resolution vessel wall MRI showed significant obliteration of the ICAs (arrows), suggesting moyamoya disease. These cumulative observations led to the final diagnosis of moyamoya disease

The initial findings on the first day revealed isolated left distal ICA occlusion, prompting considerations of atherosclerosis, moyamoya disease (MMD), or dissection (Fig. 14A). Subsequent imaging performed 8 days after onset demonstrated rapid progressive right distal ICA stenosis along with new left distal multifocal stenosis and a string-of-beads pattern (Fig. 14B). These findings initially led us to consider a possibility of RCVS superimposed on chronic left distal ICA occlusion. However, the findings 1 year after onset showed no reversal of the bilateral distal ICA steno-occlusion or the vasoconstriction in the left distal arteries (Fig. 14C). These cumulative observations led to a final diagnosis of unilateral MMD, which was confirmed by high-resolution vessel wall MRI (Fig. 14D).

MMD is prevalent in Northeast Asian populations, particularly in Japan, Korea, and China [51]. A known genetic susceptibility factor is a polymorphism in the ring finger protein 213 [52]. Previously considered primarily a pediatric disease, with 48% of cases occurring in individuals under the age of 10 years, MMD now shows an increasing prevalence in adults [53]. Recent studies have indicated that the peak age of onset is 45−49 years, making it essential to include MMD in the differential diagnosis of adults [53]. Diagnostic challenges arise in adult-onset cases because characteristic basal collaterals decrease with age, making diagnosis more difficult.

This case highlights the complexity of distinguishing between RCVS and MMD. A typical MMD involves the terminal segments of the ICAs, leading to compensatory basal collaterals that resemble a “hazy puff of smoke” and progressively affect the proximal ACA and MCAs [54–56]. On high-resolution MRI wall imaging, MMD is characterized by negative remodeling (i.e., reduced outer diameter of the affected vessels) and concentric gadolinium enhancement of the active lesion [57, 58].

In Case 11, the differential diagnosis was challenging due to unilateral involvement at baseline imaging and rapid progression in the second imaging. Although bilateral presentation is more common, unilateral moyamoya vasculopathy is observed in up to 18% of patients with MMD, and recent studies have shown contralateral progression in up to 78.6% of unilateral cases [59–62]. Unilateral MMD is more prevalent in adults, and collateral vessel development is less pronounced than that in younger patients [62–65].

In a study from the Stanford University Medical Center, seven out of 18 patients (38.9%) showed angiographic progression to bilateral disease within an average of 12.7 months, with most cases requiring surgical intervention and primarily affecting adults [61]. Koruda et al. reported 15 patients with worsening unilateral MMD, and the earliest interval for detecting worsening was 1 month [66]. A surprisingly rapid progression (8 days) in Case 11, along with the postpartum setting, raised a strong suspicion of RCVS or delayed vasospasm associated with PRES rather than MMD. However, prolonged observation and high-resolution MRI suggested a diagnosis of MMD.

### Vasospasm as a complication of subarachnoid hemorrhage

#### Case 12

A 23-year-old female presented with episodes of excruciating pain, peaking at a Numeric Rating Scale of 10/10 within 1 min while walking, occurring 20 days prior to her visit. She experienced two similar episodes of nausea, vomiting, and pain radiating from the head, neck, and back. Despite receiving analgesic treatment, the patient continued to have a persistent mild headache that progressively worsened and was unresponsive to pain medications, prompting her to seek medical attention.

**Fig. 15:**
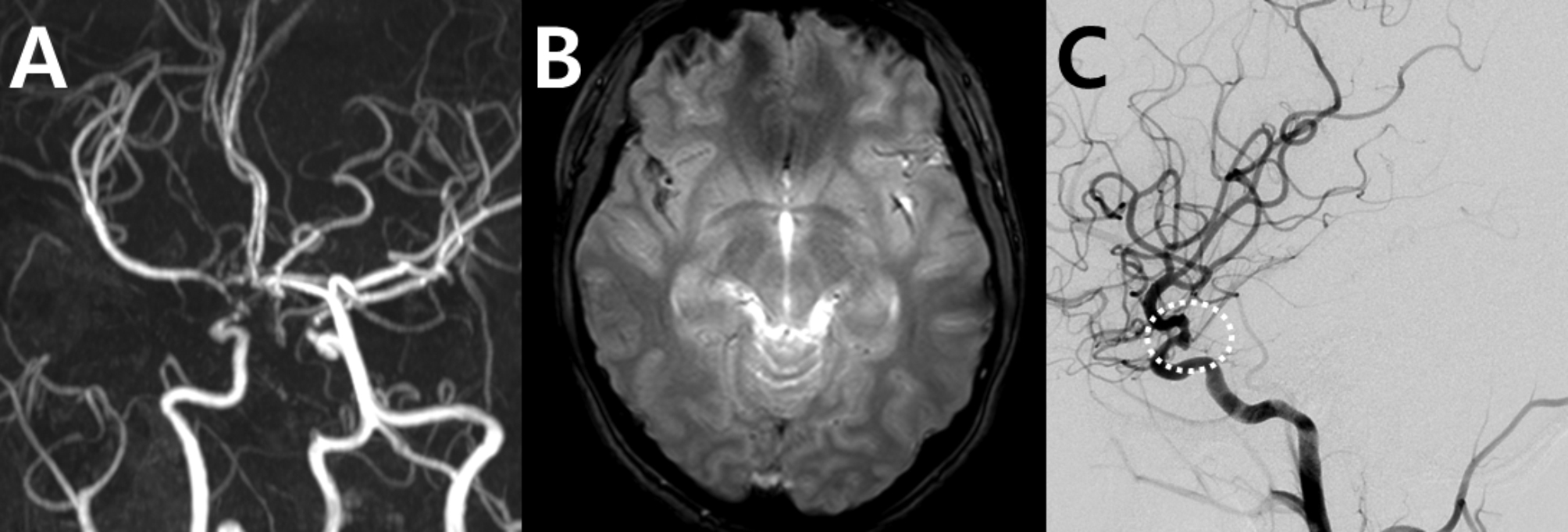
Mimickers of reversible cerebral vasoconstriction syndrome: aneurysmal subarachnoid hemorrhage and vasospasm (case 12). A 23-year-old female who had severe headache episodes was initially misdiagnosed with RCVS. (**A**) Magnetic resonance angiography (MRA) shows severe stenosis in the right distal ICA and M1 segment of the ACAs. (**B**) A small amount of subarachnoid hemorrhage is observed along the right Sylvian fissure in the gradient-echo image. (**C**) Catheter angiography reveals a ruptured aneurysm over the right distal ICA aneurysm (dotted circles), requiring emergency coil embolization

An initial CT scan was performed to rule out SAH, but the result was negative. Given the unusual localization of severe stenosis in both distal ICAs and M1 segments of the ACAs on MRA (Fig. 15A) and the presence of a small amount of hemorrhage along the Sylvian fissure (Fig. 15B), SAH with associated vasospasm was considered a potential differential diagnosis over RCVS.

To further clarify the diagnosis, CSF analysis was performed, revealing xanthochromia, consistent with the presence of red blood cells, indicative of SAH. This finding led to follow-up catheter angiography, which confirmed a ruptured aneurysm in the right distal ICA (Fig. 15C), requiring emergency coil embolization. This case underscores the importance of a thorough, stepwise diagnostic approach to differentiate SAH from RCVS, especially when atypical radiological features are present.

The differential diagnosis of vasospasm after aneurysmal SAH and RCVS with SAH can be challenging because patients can present with severe headaches, brain hemorrhage, and potential neurologic deficits. However, each type has distinct characteristics. In aneurysmal SAH, hemorrhage typically occurs in the basal cisterns or Sylvian fissure, while it is commonly found in cerebral convexity in RCVS [67, 68]. Vasoconstriction in SAH is located mainly on the bleeding site, often unilateral, and correlated with the amount of hemorrhage [69]. In contrast, RCVS involves diffuse vasoconstriction, usually bilateral, with severity disproportionate to the amount of hemorrhage (i.e., vasoconstriction is more severe and widespread, while the amount of SAH is often small) [70]. Regarding timing, SAH-related vasoconstriction peaks between 4 and 14 days after onset, while vasoconstriction in RCVS is already pronounced at onset in cases complicated by SAH [71]. Neurological deficits are more pronounced in SAH, often worsening with vasospasm, while they are transient in the majority of patients with RCVS [72]. Approximately 70% of patients with SAH experience headache, with a thunderclap pattern as the primary symptom in 50% of cases [73–76]. Headache in SAH can persist and commonly accompany neck rigidity, while the headache associated with RCVS typically presents as a thunderclap at onset, is often self-limiting, and is triggered by unique provocating factors such as urination, showering, and bending. Both diseases can be triggered by physical exertion, sexual activity, intense emotions, and Valsalva maneuvers [77–79].

### Primary angiitis of the Central Nervous System

RCVS and primary angiitis of the central nervous system (PACNS) are often considered differential diagnoses of cerebral arteriopathies [80]. This topic will be further explored and debated separately in this special collection.

Several clinical and imaging findings can differentiate between these two conditions. Clinically, RCVS often begins with a thunderclap headache and a recurrent thunderclap headache [81]. In contrast, PACNS rarely presents with a thunderclap headache; instead, it manifests itself as a gradual-onset, dull, and progressive headache [82]. Neurological deficits in RCVS are often transient, while in PACNS, they are common and persistent, frequently accompanied by cognitive and emotional impairments associated with encephalopathy, often including hemiparesis or aphasia [83, 84].

RCVS involves multiple arteries with a severe and relatively symmetric vasoconstriction and a “sausage-on-a-string” appearance [4]. PACNS may show normal angiographic findings or less severe and symmetric vasoconstriction [85]. Infarcts in the PACNS are located in multiple territories, including the deep gray matter and brainstem, and brain MRI findings are almost always abnormal [80]. In RCVS, infarcts are less common and typically small or wedge-shaped, affecting hemispheric border zones rather than deep structures; brain MRI can be normal in uncomplicated cases [80]. Hemorrhage patterns also differ; PACNS more commonly involves parenchymal hemorrhage, whereas RCVS more commonly involves convexity SAH [81]. PRES is very rare in PACNS, but not uncommon in RCVS [86]. High-resolution vessel wall MRI shows strong, concentric enhancements of the affected arterial walls in the PACNS, while RCVS shows a normal or mild enhancement, concentrically or eccentrically [87, 88].

In summary, RCVS is characterized by a thunderclap headache, transient neurological deficits, and a monophasic course, while PACNS presents with persistent symptoms, infarctions, and more severe brain parenchymal lesions. Imaging plays a vital role in diagnosis, and high-resolution vessel wall MRI and clinical history can be helpful for differentiation.

## Imaging the blood brain barrier breakdown for the differential diagnosis of reversible cerebral vasoconstriction syndrome

As discussed, imaging findings of vasoconstriction alone may be unsuccessful in diagnosing RCVS because vasoconstriction in RCVS can have a broader spectrum than its typical multifocal vasoconstriction-vasodilation pattern, and differential diagnosis with a variety of vasculopathies can be challenging. HR-vwMRI can help in the differential diagnosis as an ancillary test for selected cases of proximal arterial involvement to differentiate RCVS from conditions such as dissection, ICAS, MMD, and PACNS. Its use is limited in evaluating distal arteries due to their thin walls, which are smaller than the voxel resolution of HR-vwMRI, and the resulting low signal-to-noise ratio [89]. Our group showed that the pathophysiology of RCVS includes BBB breakdown, which can be visualized using contrast-enhanced fluid-attenuated inversion recovery (FLAIR) MRI by detecting BBB disruption through hyperintense CSF when gadolinium leaks into CSF and parenchyma, shortening T1 and reducing signal suppression [90–93]. CE-FLAIR imaging is also considered an ancillary test, but its routine use in clinical practice is strongly recommended as it provides valuable information about small arteriolar and capillary involvement which cannot be visualized in current angiographic imaging. It further assists in differentiating RCVS from conditions such as ICAS, MMD, and dissection. Imaging findings in RCVS typically peak at 1–2 weeks but can still be detected up to 1 month after symptom onset, offering an extended diagnostic window [94].

**Fig. 16:**
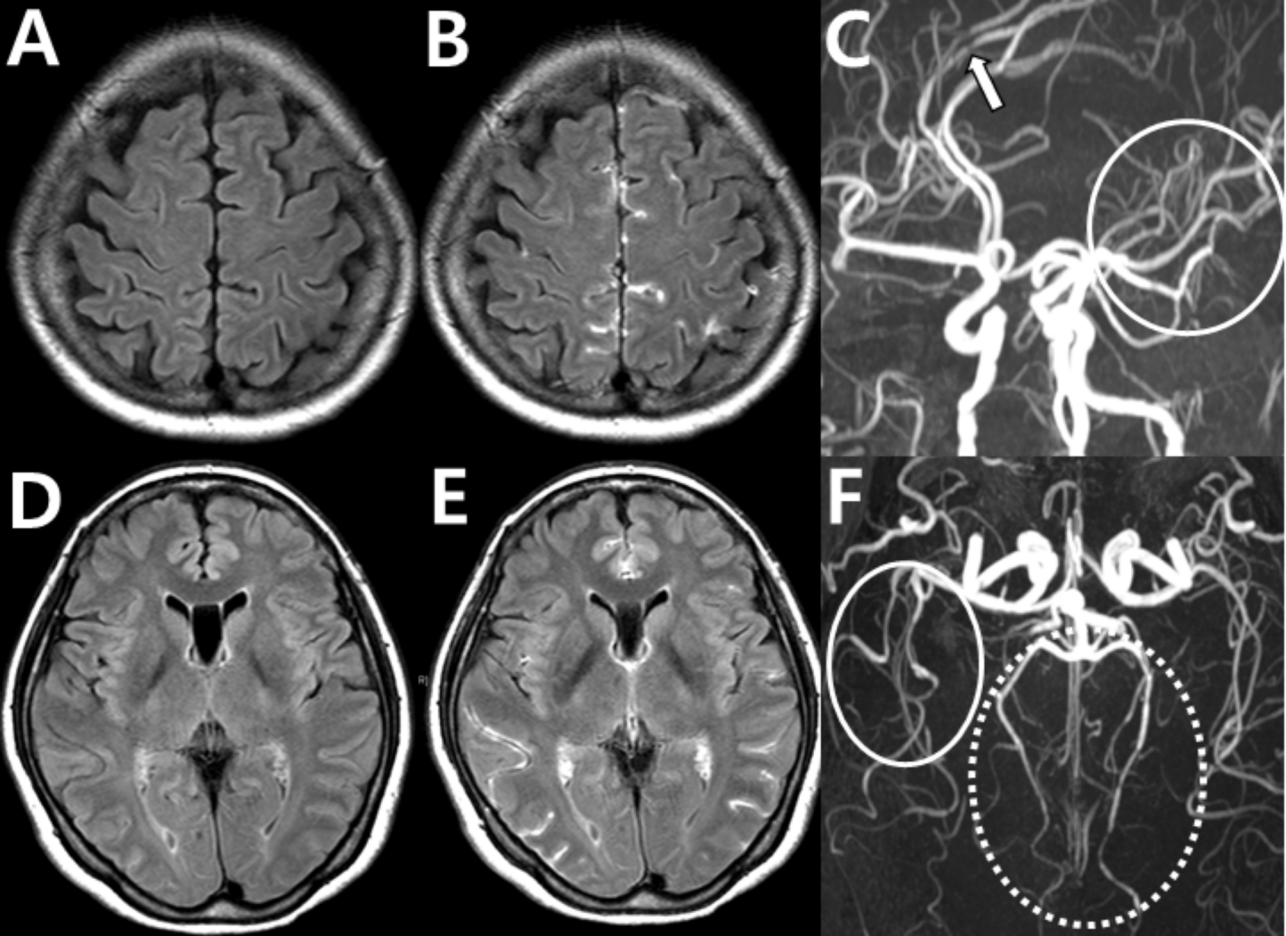
Blood-brain barrier disruption in reversible cerebral vasoconstriction syndrome. BBB breakdown is observed using contrast-enhanced FLAIR imaging in a 56-year-old female with RCVS (case 2). (**A, D**) FLAIR images without contrast. (**B, E**) The same sections with contrast-enhanced FLAIR MRI show BBB disruption through hyperintense CSF. (**C, F**) MRA shows typical multifocal steno-dilatation in the ACA (arrow), left MCA (solid circles), and distal PCAs (dotted circles)

In our study, BBB breakdown was documented in 69% of patients with definite RCVS and 25% of those with probable RCVS but not in those with other secondary causes of thunderclap headache, underscoring its diagnostic value [93]. Subsequently, this finding was reproduced in several studies [94–96]. Chen et al. (2021) elaborated on this idea and successfully demonstrated increased BBB permeability using dynamic contrast-enhanced MRI in patients with RCVS [95, 96]. BBB breakdown in RCVS appears earlier than vasoconstriction, with a peak prevalence in the first and second weeks, while vasoconstriction peaks in the third week [94]. This explains the different timings of neurological complications associated with RCVS: hemorrhagic lesions and PRES develop earlier (1–2 weeks after onset), while ischemic stroke appears later (2–3 weeks after onset) [94, 97, 98]. Therefore, BBB breakdown can serve as a useful marker for the early diagnosis and prediction of neurological complications in RCVS [93]. However, caution is necessary when interpreting BBB breakdown, as BBB disruption can also occur in conditions other than RCVS, such as SAH, posterior reversible encephalopathy syndrome, and cerebral infarction [22, 93, 99], and sulcal hyperintensity in CE-FLAIR can also be caused by leptomeningeal enhancement from infectious meningitis or leptomeningeal carcinomatosis. Therefore, a complete picture of the clinicoradiological presentation and time course should be considered when diagnosing RCVS (Fig. 16).

## Summary and Suggested Diagnostic Flow

As shown in our real-world cases, RCVS diagnosis poses significant challenges due to its varied clinical presentation and limitations in imaging modalities. The diagnosis is often complicated by the fact that angiography can appear normal in the early stages of RCVS and the interpretation of MRA can be difficult due to potential artifacts or focal or diffuse lesions. Differential diagnoses such as atherosclerosis, arterial dissection, MMD, vasospasm after SAH, and PACNS require a thorough evaluation (Table 1). RCVS should be considered because imaging findings can vary beyond the radiological peak, and even when imaging findings are normal or atypical, it should still be included in the differential diagnosis.

**Table 1:** Differential diagnoses of reversible cerebral vasoconstriction syndrome

	Reversible Cerebral Vasoconstriction Syndrome	Intracranial Atherosclerosis	Intracranial Arterial Dissection	Moyamoya Disease	Subarachnoid Hemorrhage	Primary Angiitis of the Central Nervous System
Clinical Symptoms	Recurrent, sudden-onset thunderclap headaches triggered by sexual activity, exertion, Valsalva maneuvers	Often asymptomatic until significant blockage	Variable symptoms; may present with headache or focal neurologic deficits	Transient ischemic attack, stroke, headache during exertion, stress, fever	Persistent thunderclap headache, neck stiffness or focal neurologic deficits	Gradual-onset, dull headache with persistent neurological deficits, cognitive changes
MRI findings						
Angiographic findings	“String of beads” appearance with reversible vasoconstriction	Less severe, less symmetric; segmental dilatation rare	Typically affects a single artery	“Puff of smoke” collateral pattern; progressive stenosis of internal carotid artery terminal segments	Vasoconstriction near the bleeding site, often unilateral and peak between 4–14 days after onset	Normal or less severe, symmetric vasoconstriction
Parenchymal findings	Hemorrhage usually in convexity; blood brain barrier breakdown shown in contrast-enhanced fluid-attenuated inversion recovery MRI	May show ischemic changes in advanced stages	May include ischemia or hematoma in advanced stages	May include chronic ischemia, infarctions, encephalomalacia, or hemorrhage	Hemorrhage in basal cisterns or Sylvian fissure	Multiple infarcts in deep gray matter or brainstem
HR-vwMRI findings	Mild or no enhancement	Eccentric atheromas, positive remodeling, plaque enhancement	Eccentric narrowing, intimal flaps, intramural hematoma	Negative remodeling, concentric enhancement	Useful in identifying rupture site of aneurysmal subarachnoid hemorrhage	Concentric enhancement
Differential Point	Diffuse, bilateral vasoconstriction with reversibility which rarely involves internal carotid artery	HR-vwMRI findings	Number of vessels involved and HR-vwMRI findings	Angiographic and HR-vwMRI findings	Hemorrhage site and vasoconstriction timing	Parenchymal and HR-vwMRI findings

**Abbreviations: MRI, magnetic resonance imaging; HR-vwMRI, high-resolution vessel wall magnetic resonance imaging;

As shown in these cases, relying solely on MRA has intrinsic limitations. A thorough clinical evaluation, and follow-up imaging, with additional consideration of high-resolution vessel imaging can help with the diagnosis. Further to these, BBB breakdown is another important diagnostic and pathophysiologic marker of RCVS, which can be documented by using a delayed contrast-enhanced FLAIR protocol after gadolinium injection [91, 92, 95, 99, 100]. A real-world study found that 69% of patients with definite RCVS, one-fourth of patients with probable RCVS, and one-eighth of patients with primary thunderclap headache had a BBB breakdown [93]. Using BBB breakdown as a complementary finding, the diagnostic rate of RCVS increased from 33 to 41% in patients with normal angiography [93]. When BBB breakdown and clinical manifestations were considered together, RCVS was diagnosed in 61% of patients with thunderclap headache compared to 40% based only on angiographic findings [93]. This technique can help in the diagnosis of RCVS in angiogram-negative patients.

**Fig. 17:**
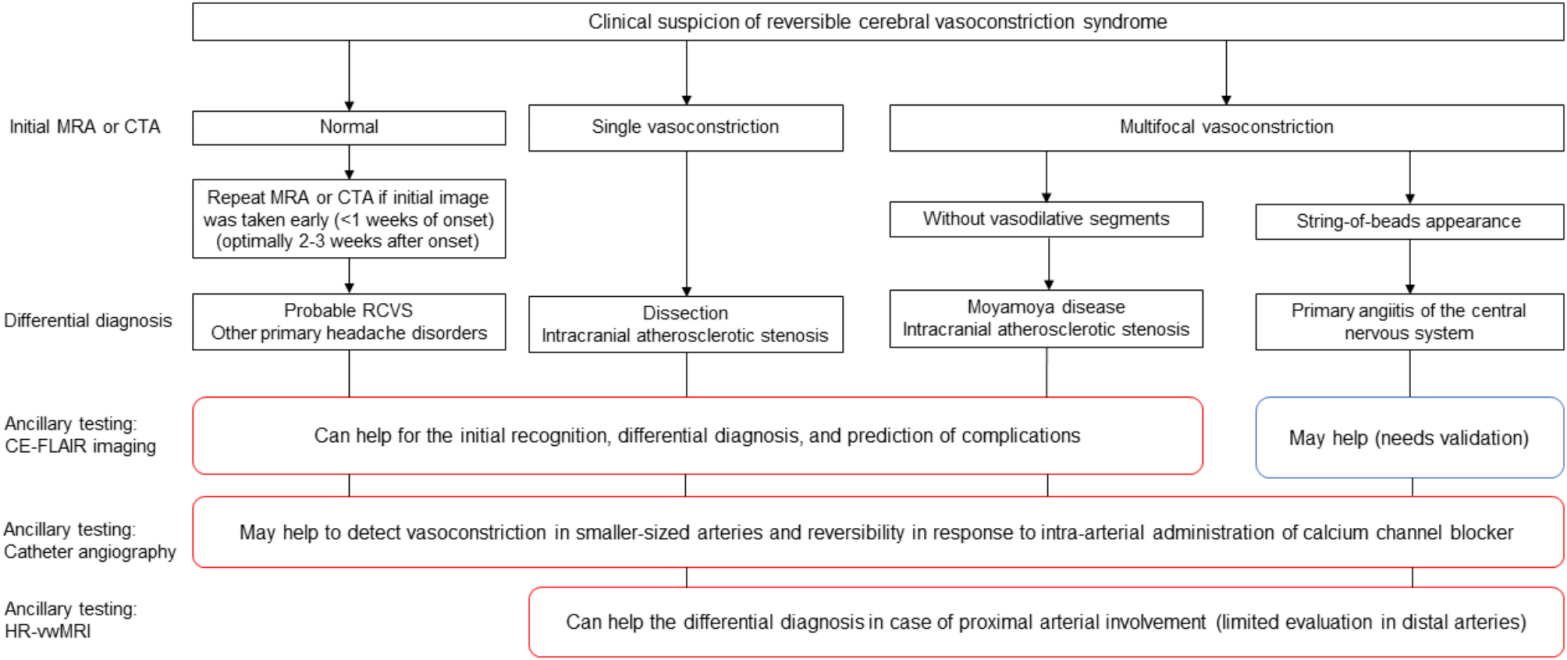
Diagnostic flow chart for radiologic evaluation and differential diagnosis in suspected reversible cerebral vasoconstriction syndrome. **Abbreviations: MRA, Magnetic Resonance Angiography; CTA, Computed Tomography Angiography; RCVS, Reversible Cerebral Vasoconstriction Syndrome; CE-FLAIR, Contrast-Enhanced Fluid-Attenuated Inversion Recovery; HR-vwMRI, High-Resolution Vessel Wall Magnetic Resonance Imaging;

Figure 17 is a diagnostic flow chart with a focus on radiologic evaluation to guide differential diagnoses and imaging options for RCVS. MRA and CTA are both viable options for initial imaging; however, MRA is recommended as the initial imaging modality due to its standardized imaging parameters and superior safety profile, as it is non-invasive and avoids the risks of nephrotoxicity and radiation exposure. Repeat scans are advised 2–3 weeks after onset if the initial findings are normal and the scan was performed early (< 1 week after onset). Single lesions may indicate dissection or ICAS, while multifocal vasoconstriction without vasodilative segments suggests MMD or ICAS. A “string-of-beads” pattern raises suspicion for PACNS alongside RCVS. CE-FLAIR helps differentiate RCVS from dissection, ICAS, or MMD, although its utility in PACNS requires further validation. Catheter angiography, although invasive, may be useful across all stages, detecting vasoconstriction in smaller vessels and assessing reversibility with intra-arterial calcium channel blockers. HRMR is particularly effective for evaluating proximal arterial involvement in dissection, ICAS, MMD, and PACNS, though its application to distal arteries remains limited.

This flow chart summarizes imaging approaches and differential diagnoses for suspected RCVS. It provides guidance on the use of specific imaging modalities, including contrast-enhanced fluid-attenuated inversion recovery, catheter angiography, and high-resolution vessel wall magnetic resonance imaging. Red-outlined boxes in the flow chart indicate ancillary tests, which are not mandatory but may provide additional diagnostic information in specific clinical scenarios.

In conclusion, the diagnosis of RCVS requires a comprehensive approach that integrates clinical and imaging findings. Awareness of diagnostic challenges and differential diagnoses, combined with advances such as high-resolution vessel wall MRI and BBB breakdown assessment, can improve diagnostic accuracy and improve patient outcomes.

## Data Availability

The data used in this study consisted of patient cases and were subject to privacy protection under Institutional Review Board (IRB) regulations. Due to ethical restrictions, raw data cannot be publicly available or shared on request. Data and materials supporting this study’s conclusions are included in this article.

## Abbreviations

RCVS: Reversible cerebral vasoconstriction syndrome
MRA: Magnetic resonance angiography
TOF: Time-of-flight
ICA: Internal carotid artery
MCA: Middle cerebral artery
ACA: Anterior cerebral artery
PCA: Posterior cerebral artery
ICHD-3: International Classification of Headache Disorders, 3rd edition
BBB: Blood-brain barrier
HR-vwMRI: High-resolution vessel wall MRI
SAH: Subarachnoid hemorrhage
CE-FLAIR: Contrast-enhanced fluid-attenuated inversion recovery
PRES: Posterior reversible encephalopathy syndrome
ICAS: Intracranial atherosclerotic stenosis
MMD: Moyamoya disease
CSF: Cerebrospinal fluid
PACNS: Primary angiitis of the central nervous system
